# Phase patterning of metallic glasses through superfast quenching of ion irradiation-induced thermal spikes

**DOI:** 10.1186/s40580-023-00400-7

**Published:** 2023-11-21

**Authors:** Hyosim Kim, Tianyao Wang, Jonathan Gigax, Arezoo Zare, Don A. Lucca, Zhihan Hu, Yongchang Li, Trevor Parker, Lin Shao

**Affiliations:** 1 Los Alamos National Laboratory, Los Alamos, NM 87545 USA; 2 Department of Nuclear Engineering, Texas A&M University, College Station, TX 77843 USA; 3 School of Mechanical and Aerospace Engineering, Oklahoma State University, Stillwater, OK 74078 USA

**Keywords:** Metallic glass, Crystallization, Amorphization, Damage cascades

## Abstract

Amorphous metallic glasses (MGs) convert to crystalline solids upon annealing at a high temperature. Such a phase change, however, does not occur with the local melting caused by damage cascades introduced by ion irradiation, although the resulting thermal spikes can reach temperatures > 1000 K. This is because the quenching rate of the local melting zone is several orders of magnitude higher than the critical cooling rate for MG formation. Thus the amorphous structure is sustained. This mechanism increases the highest temperature at which irradiated MG sustains amorphous phase. More interestingly, if an irradiated MG is pre-annealed to form a polycrystalline structure, ion irradiation can locally convert this crystalline phase to an amorphous phase if the grains are nanometers in size and comparable to the damage cascade volume size. Combining pre-annealing and site selective ion irradiation, patterned crystalline-amorphous heterogeneous structures have been fabricated. This finding opens new doors for various applications.

**Supplementary Information:**

The online version contains supplementary material available at 10.1186/s40580-023-00400-7.

## Introduction

Metallic glasses (MGs) form through the rapid quenching of a molten alloy at a rate exceeding the critical cooling rate (R_c_) [1–3]. Composition engineering has been successfully used to increase the viscosities of MGs, which decreases the critical cooling rates that are needed to fabricate bulk MGs of large volume for practical applications [1–3]. MGs exhibit exceptional strength and elasticity [2–4], but have limited plasticity due to shear localization. Under tensile loading, MGs show almost no macroscopic ductility [5]. One way to improve ductility is by introducing ductile crystalline dendritic particles to promote shear band initiation and to arrest shear bands to avoid catastrophic failure [5]. However, it’s essential for the microstructural length scale of the particles to match the characteristic dimension of a crack tip’s plastic zone [5].

Nanocrystals can be easily introduced into MGs through thermal annealing at temperatures above the glass transition temperature (T_g_). The effects on ductility, however, are controversial, with both embrittlement and toughening observed [6–12]. The size, volume fraction, and ductility of the nanoparticles are critical for tuning the global ductility [13]. From the perspective of process control, thermal annealing presents challenges in process control due to the lack of control on nucleation sites and nuclei densities. Furthermore, in many MG systems, the activation energies of crystal nucleation are higher than that of crystal growth [14–16], which makes crystal volume control difficult due to easy crystal growth at temperatures high enough to initiate crystal nucleation. As a result, it is difficult to use thermal annealing for ductility improvement. Other methods such as bending [16], ball milling [17, 18], indentation [19], and pressure [20], can induce nanocrystals, but typically in a destructive manner. Ion irradiation or electron irradiation has also been used to induce crystallization [21–23], but results vary, with nanocrystallization observed in some cases and not in others [24]. We believe that the observed differences in results are a consequence of the irradiation conditions used and the various mechanisms involved. Specifically, for ultra-high current ion irradiation, the total energy deposited at the surface can be high enough to induce thermal annealing, and therefore ion irradiation acts as a heating source. Evidence of such an effect is that crystallization occurs throughout the specimen, including regions well beyond the projected range (R_p_) of the ions. For intermediate beam current ion irradiation at high fluence, beam heating is not significant but crystal nucleation is still possible due to the enhanced atomic mobility resultant from both the thermal effects and athermal effects of irradiation. The atomic mobility,$D$ is calculated by $D={D}_{0}\text{e}\text{x}\text{p}(-E/kT)\text{e}\text{x}\text{p}(\varDelta {V}_{FV}/\varDelta {V}_{0})$ [25, 26], where ${D}_{0}$ is a constant, $E$ is migration energy, $k$ is Boltzmann constant, $T$ is temperature, $\varDelta {V}_{FV}$ is the excess free volume and $\varDelta {V}_{0}$ is an activation volume. Damage cascades create local density fluctuations and $\varDelta {V}_{FV}$ changes. In combination with the local heating effect, crystal nucleation is promoted. Microstructural changes under such conditions exhibit multiple stages. First, small ordered atoms diffuse into precipitates. Second, large crystals grow within the precipitates [27]. There is no linear correlation between ion fluence and crystal density, and no evidence that the damage cascade region is directly converted to crystals.

In the present study, we limit our investigation to low beam current irradiation in which ion-solid interactions are dominated by single damage cascade effects. The average amount of power deposited by the beam is ignorable under low beam current conditions. The key finding, as to be discussed, is that instead of directly converting the local region into a crystalline phase, ion damage cascades sustain the amorphous phase, regardless of the initial phase conditions (whether the MG starts as an amorphous material or as an annealed crystalline material). In the present study we irradiate a pre-annealed polycrystalline MG for site selective amorphization. The study demonstrates that it is critical to control the grain size of the annealed material. Such an effect occurs over a very large irradiation temperature range (up to ~ T_g_). Different from other crystallization methods (deformation, indentation, annealing, and ion irradiation of an amorphous MG), the method can precisely control the crystalline phase size and volume in a patterned heterogeneous structure. The patterning is achieved through lithography and masking techniques. The method can be easily adopted to tune the mechanical properties with crystal morphology satisfying both the length scale and volume required for ductility optimization.

## Results and discussions

**Fig. 1:**
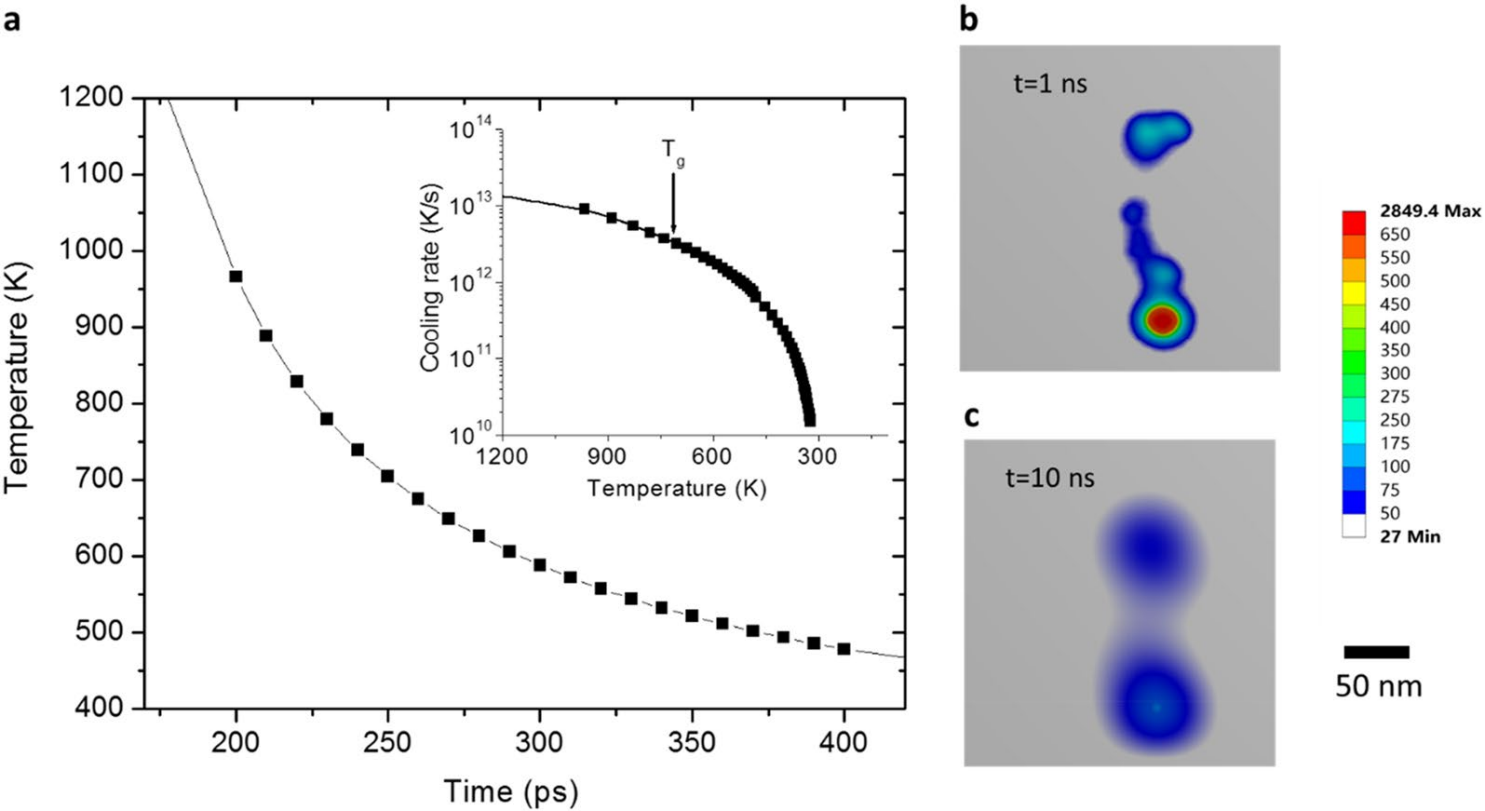
**a** Results of a simulation showing superfast quenching of one damage cascade core produced by bombardment of 3.5 MeV Cu ions. **b, c** Temperature mapping of the damage cascade core at 1 ns and 10 ns after ion bombardment. The modeling is obtained by combining the SRIM code and finite element analysis

Although thermal spike temperatures in a damage cascade can initially exceed 1000 K, local heat dissipation is too fast to allow for crystal nucleation. Figure 1a shows the temperature change in a single damage cascade created by 3.5 MeV Cu ion bombardment of a Ti_40_Cu_29_Zr_10_Pd_14_Sn_2_Si_5_ MG, simulated by combining a Monte Carlo simulation (SRIM) of damage cascade creation and finite element analysis of thermal quenching (further details are shown in Additional file 1: Figure S1). At 200 ps, the core temperature reaches about 1000 K. At 247 ps, the temperature reduces to 713 K, which is the glass transition temperature (T_g_). The inset of Fig. 1a shows the cooling rate. The average cooling rate at 713 K is 3.5 × 10^12^ K/s, which is 7 orders of magnitude higher than the critical cooling rate R_c_ for MG formation. The critical R_c_ of Ti-Cu based MGs is on the order of 1 × 10^5^ K/s [28]. The significance lies in the fact that the damage cascades cannot directly induce local amorphous-to-crystalline phase changes because the thermal quenching of the damage cascade region is too fast. As a result, the molten MG retains its glassy state upon cooling to room temperature. Figure 1b and c show temperature contours of the thermal spikes from the damage cascades, at 1 ns and 10 ns, respectively. The melting point of the Ti-Cu system is about 980 °C [12]. Using this temperature as a boundary, the temperature contours of the damage cascades suggest a melting zone of 30 to 50nm in size due to the evolution of the thermal spikes [see Additional file 1: Fig. S2].

**Fig. 2:**
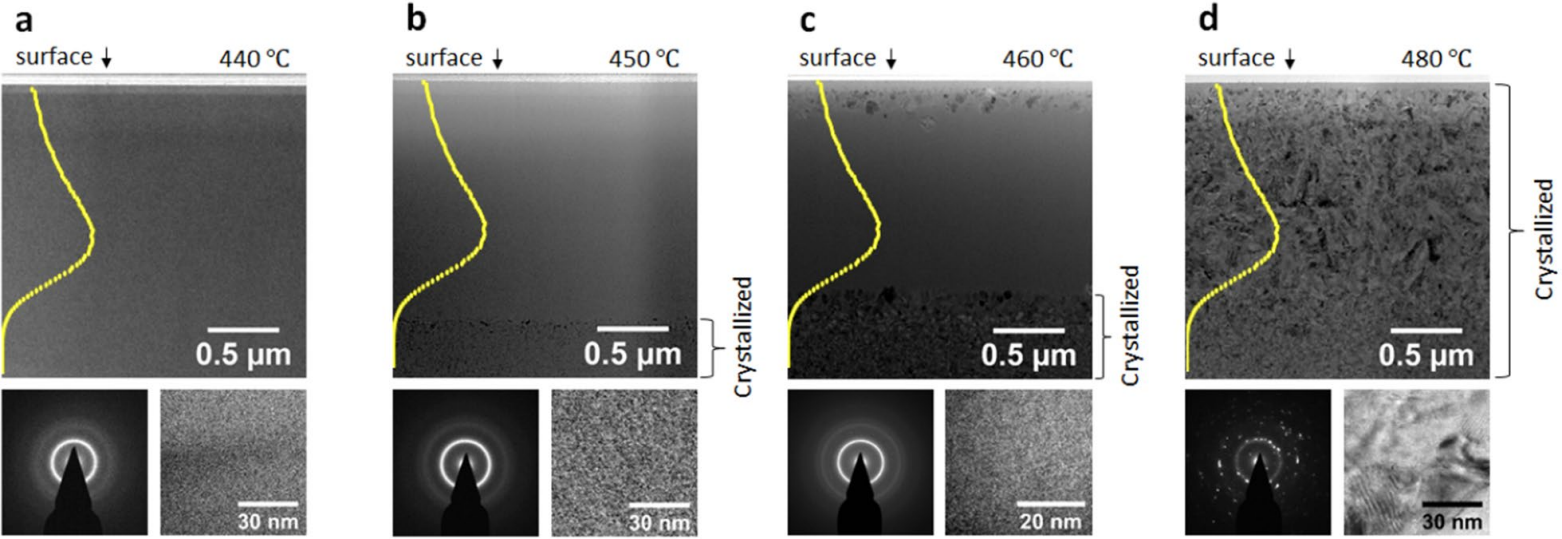
**a–d** Cross sectional TEM micrographs, localized diffraction patterns, and HRTEM images of an MG irradiated by 3.5 MeV Cu ions to a fluence of 1 × 10^16^ ions/cm^2^ at 440 °C, 450 °C, 460 °C, and 480 °C, respectively. Superimposed are SRIM damage profiles. All the diffraction patterns and HRTEM micrographs were collected within the damage zone (depth < R_P_)

As evidence that the damage cascades cannot directly convert an MG to a crystalline solid, Fig. 2a-d show cross sectional transmission electron microscopy (TEM) micrographs of a Ti_40_Cu_29_Zr_10_Pd_14_Sn_2_Si_5_ MG irradiated by 3.5 MeV Cu ions to a fluence of 1 × 10^16^ ions/cm^2^ at temperatures of 440, 450, 460 and 480 °C, respectively. Irradiation at 440 °C does not induce crystallization, as indicated by both the amorphous ring diffraction pattern and the lack of ordered structure in the high resolution TEM (HRTEM) micrograph shown in Fig. 2a. Superimposed in each of the TEM micrographs is the SRIM calculated displacement-per-atom (dpa) profile. The projected range (R_P_) of the 3.5 MeV Cu ions is about 1.6 μm. No crystallization or precipitation is observed at depths beyond the R_P_ (Fig. 2a), suggesting that the original glassy state is stable at this temperature. At an irradiation temperature of 450 °C, deep regions (> R_P_) are crystallized (Fig. 2b) due to the thermal annealing effect only. But within the R_P_, the amorphous phase is sustained, as further evidenced by the localized diffraction pattern and HRTEM micrograph. At a higher temperature of 460 °C, the deep crystalline region (depth > R_P_) has noticeable grain growth. But the irradiated region (< R_P_) still remains amorphous, except for the very near surface region where a few nanocrystals form. Scanning TEM and an EDS line scan show Cu loss and Ti enrichment within 80 nm below the surface, which is expected to change the local irradiation response and promote local crystallization [see Additional file 1:  Fig. S3]. At the highest temperature of 480 °C (Fig. 2d), the irradiated region is fully crystallized. These studies show that the formation of a heterogeneous structure using ion irradiation requires a very narrow temperature window. For example, T ≤ 440 °C results in a full amorphous phase, while T ≥ 480 °C results in a crystalline phase.

The composition changes near the surface could be caused by (1) preferential atomic loss due to ion sputtering; (2) elemental segregation towards the surface; (3) minor surface contamination during irradiation. It is worth noting that ion irradiation-induced free volumes are positive and substantial near the surface region [29], which can promote local crystallization even without composition changes. Hence, the formation of an ultra-thin crystalline layer, as observed in Fig. 2c, is complicated and could be a combination of several mechanisms, both from irradiation and temperature effects.

For irradiation at 480 °C, the crystallization shows noticeable differences at different depths. In the near-surface region, there is a continuous crystallized layer with a thickness of about 8 nm. Within the ion irradiated region and beneath the surface crystallized layer, large crystals (> 50 nm) are observed. The crystal sizes increase with increasing local damage levels and peak at the depth corresponding to the peak damage location. The crystalline size begins to reduce after the damage peak. At depths outside of the ion irradiation region, crystalline sizes are significantly reduced. Clearly, ion irradiation at a high temperature such as 480 °C promotes crystallization. The grain growth is promoted due to enhanced atomic mobility under ion irradiation. For defect-assisted diffusion, atomic mobility increases with increasing point defect supersaturation levels.

Since the damage cascades can melt and sustain the amorphous phase at an irradiation temperature up to T_g_, the temperature window to form heterogeneous structures is significantly wider (increased from a window width of ~ 10s °C to ~ 440 °C) if the starting material is polycrystalline (annealed MG), instead of as-spun material. This method consists of thermal annealing the MG to form nanograined structures, prior to irradiation. This further requires that the composition of the local melting zones within the damage cascades is close to that of the as-spun MG. If the composition is significantly different, as would be expected if the MG grows into large crystal domains of different crystalline phases, local critical cooling rates will be significantly changed and increased up to a rate like “traditional” metals, e.g., pure Ti, such that irradiation can no longer “freeze” the amorphous phase upon cooling. If the grain size of the annealed MG is comparable to a damage cascade volume, atomic mixing and local melting upon damage cascade creation can average the composition of neighboring domains, leading to a composition close to the original MG. Hence the irradiated region will have the capability to form glassy states. The above hypothesis plays a key role in our proposed methodology. The hypothesis is supported by our experimental studies, as will be explained below.

**Fig. 3:**
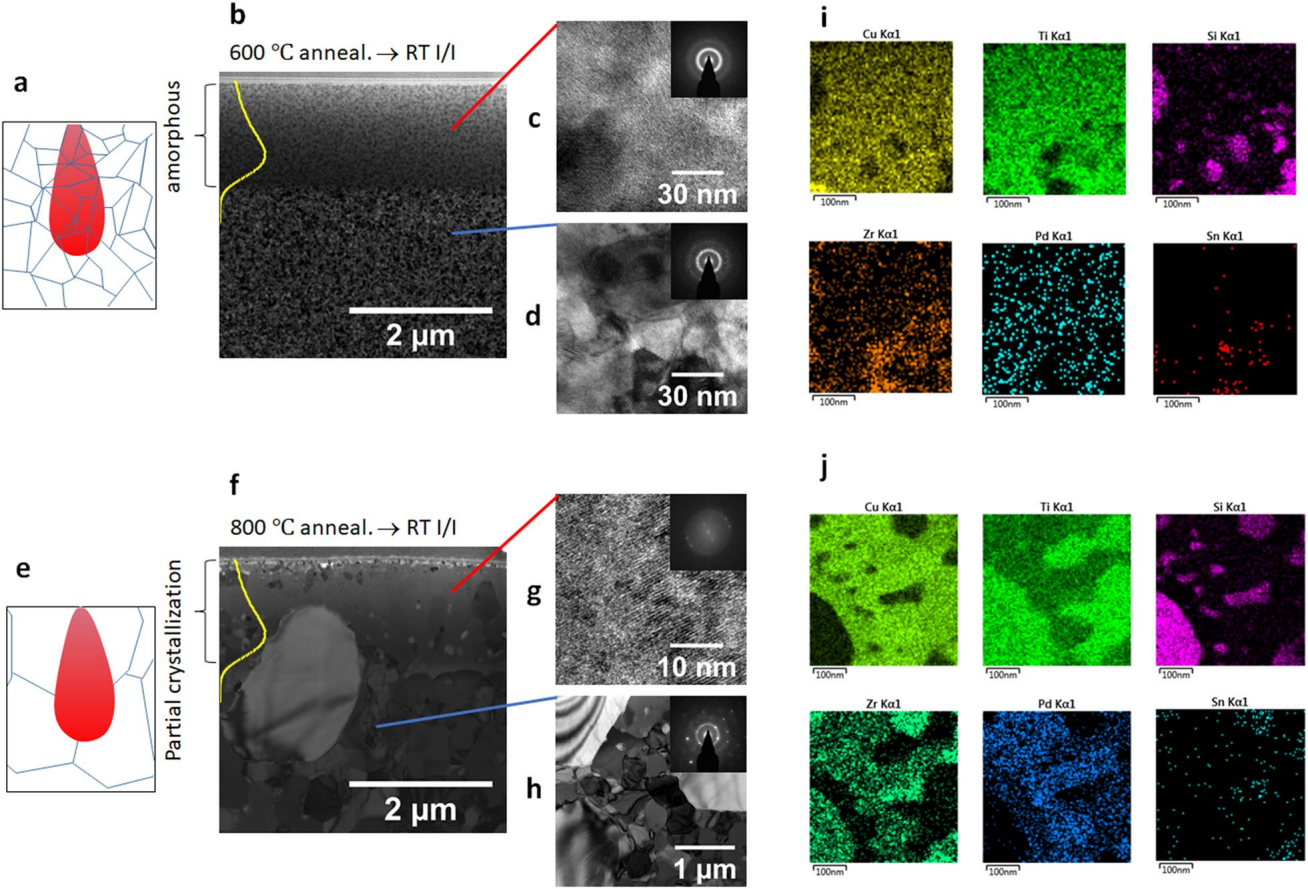
**a−d** Damage cascade schematic, cross sectional TEM micrograph, HRTEM/diffraction pattern within R_p_, and HRTEM/diffraction pattern beyond R_p_ of an annealed (600 °C for 2 h) + irradiated (3.5 MeV Cu at room temperature, 1 × 10^14^/cm^2^) MG. **e**–**h** Damage cascade schematic, cross sectional TEM micrograph, HRTEM/diffraction pattern within R_p_, and HRTEM/diffraction pattern beyond R_p_ of an annealed (800 °C for 4 h) + irradiated (3.5 MeV Cu at room temperature, 1 × 10^14^/cm^2^) MG. **i** EDS mapping, collected within R_p_, for the specimen shown in f. **j** EDS mapping, collected beyond R_p_, for the specimen shown in f. SRIM dpa curves are superimposed in b and f

Figure 3a and e are schematics of damage cascade melting/mixing in nanograins and large grains, respectively. Figure 3b and f are TEM micrographs of radiation-induced structural changes of a pre-annealed MG with nanograin and large grain size, respectively. Both are annealed at T > T_g_, prior to irradiation, to form crystalline grains. For low temperature annealing at 600 °C for 2 h, the average grain size is about 30 nm (Fig. 3d). After ion irradiation, the irradiated region (depth < R_P_) becomes fully amorphous, while deeper regions (depth > R_P_) are crystalline. Damage cascade mixing (with a typical size of 30 to 50 nm) over several small grains (as shown in Fig. 3a) enables the irradiation response to be comparable to that of an as-spun MG. The resulting irradiated region is completely amorphous, as shown by the HRTEM image and diffraction pattern (Fig. 3c). For a crystalline MG annealed at a higher temperature of 800 °C for 4 h, the resulting grain sizes are much larger, measured as 435 ± 124 nm, and are observed for depths > R_P_ (Fig. 3h). The majority of the irradiated region within the R_P_ is found to be crystalline (Fig. 3g). Only at a few very small regions, is an amorphous phase observed (see Additional file 1:  Fig. S4). X-ray diffraction shows that the crystalline phases are a combination of faced-centered cubic Ti and two intermetallic compounds, body-centered tetragonal Cu_3_Ti_3_ and hexagonal TiPd_3_ (see Additional file 1:  Fig. S5). Figure 3i and j compare the Energy-Dispersive X-ray Spectroscopy (EDS) elemental mapping results within R_P_ and beyond R_p_ for the specimen shown in Fig. 3f. Large precipitates are observed beyond R_p_. The precipitate size is smaller within R_p_, suggesting a collision mixing effect.

**Fig. 4:**
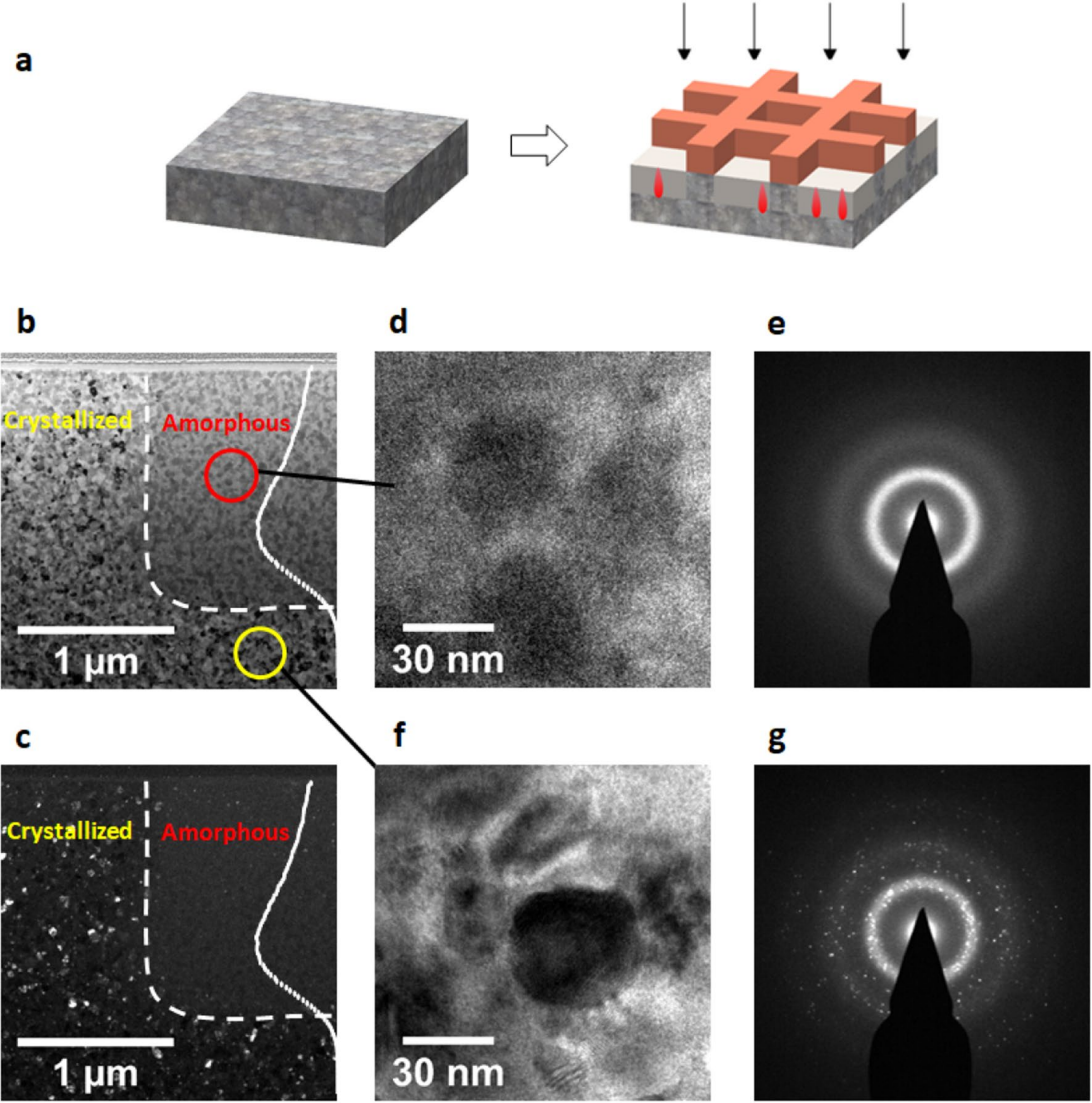
**a** Schematic of a MG after annealing at 600 °C for 2 h to form nanograins and subsequently irradiated by 3.5 MeV Cu ions to a fluence of 1 × 10^14^ ions/cm^2^ at room temperature through a mask. **b**, **c** Cross sectional TEM bright field and dark field micrographs that show patterned region. **d**, **e** HRTEM image and localized diffraction pattern collected from the irradiated region, as marked by the red circle in **b**. **f**, **g ** HRTEM image and localized diffraction pattern collected from the region beyond R_p_, as marked by the yellow circle in **b**

A technique for phase patterning is therefore proposed, as shown in the schematic of Fig. 4a, using the procedure of (1) thermal annealing at 600 °C for 2 h to form a polycrystalline MG with nanometer size grains; (2) using a TEM grid as a mask for irradiation protection; (3) irradiation at room temperature (3.5 MeV Cu, 1 × 10^14^/cm^2^) to convert the crystalline phase of the unmasked region to an amorphous phase. Figure 4b shows a cross sectional TEM micrograph of the annealed and irradiated MG. Figure 4c is the dark field image of the same region. The white dashed line refers to the boundary of the irradiated and un-irradiated regions. HRTEM micrograph (Fig. 4d) and localized diffraction pattern (Fig. 4e), both collected within the irradiated region (as indicated by the red circle in Fig. 4b) show that the irradiated region is fully amorphous. Note that the box-like amorphous region is 1.6 μm deep, which is consistent with the SRIM calculated R_p_. In the region > R_p_ (as indicated by the yellow circle in Fig. 4b), both the HRTEM micrograph (Fig. 4f) and diffraction pattern (Fig. 4g) show nanocrystal formation. As further evidence, in the dark field TEM image (Fig. 4c), the unirradiated region is featured with nanocrystals which appear with white contrast while the irradiated region appears dark.

Additional experiments were conducted to investigate the influence of ion fluence in the ion amorphization step. No noticeable ion fluence effect is observed for room temperature ion irradiation. Different fluences, ranging from 1 × 10^14^/cm^2^ to 1 × 10^17^/cm^2^, all yield similar structural outcomes. However, when ion irradiation is carried out at a higher temperature of 250 °C, significant changes occur. The amorphous regions become narrower with increasing fluence. This phenomenon is attributed to the growth of grains in the unirradiated portion, which has not undergone direct irradiation but has experienced thermal annealing during prolonged irradiation. The growth of these grains promotes crystallization and shifts the interface between the amorphous and crystalline regions toward the ion-irradiated zone, resulting in a reduction in the width of the amorphous zone. This complex behavior suggests that room temperature irradiation is the preferred choice when aiming to achieve specific phase patterning.

**Fig. 5:**
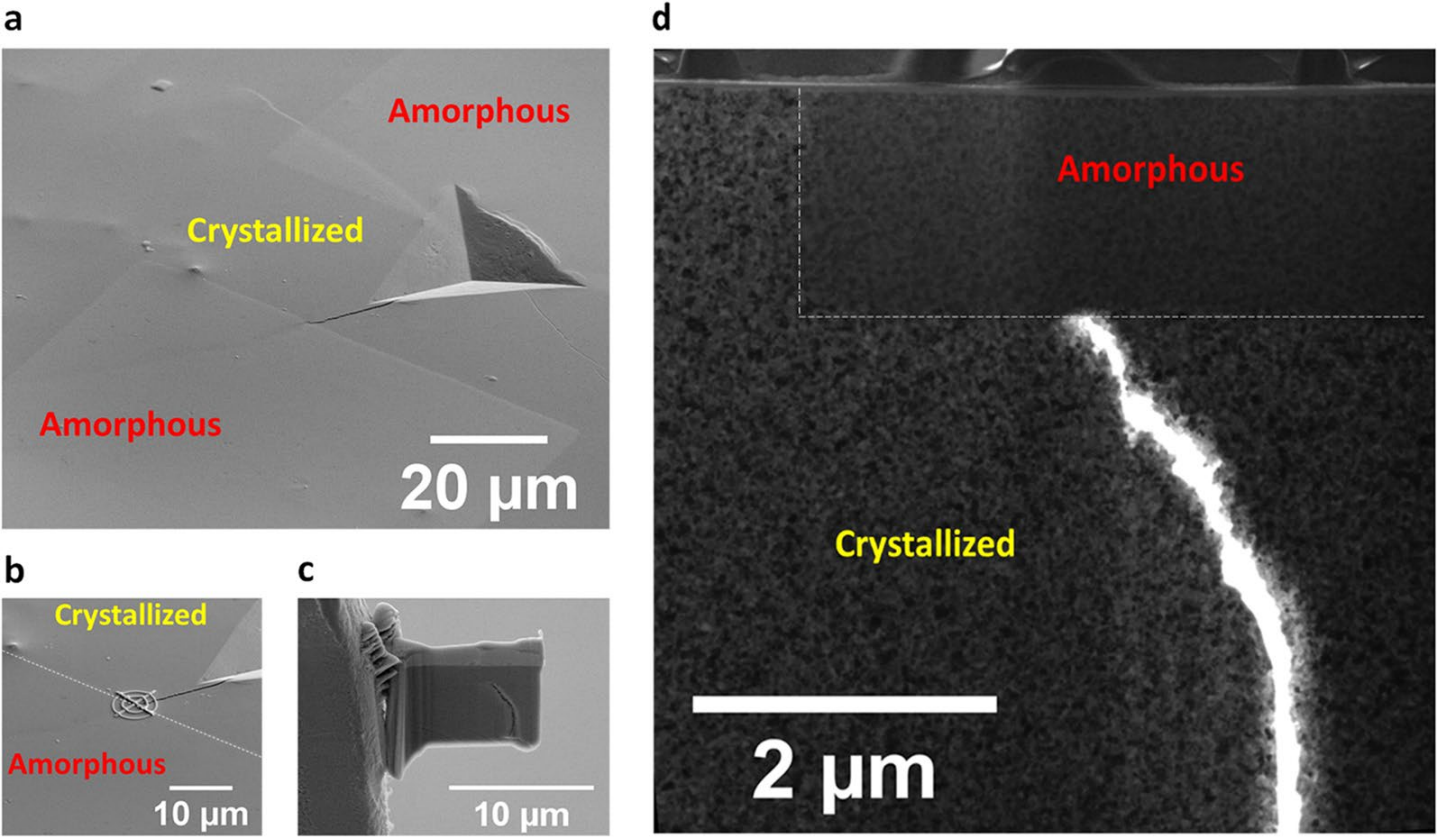
**a** SEM image of an annealed + irradiated MG, after indentation. Specimen is first annealed at 600 °C to induce nanocrystallization, and then covered by a TEM grid for patterned irradiation by 1 × 10^14^/cm^2^, 3.5 MeV Cu ion irradiation at room temperature. Indentation is performed on the crystallized region, which is the region protected by the TEM grid from ion irradiation. Indentation induces crack propagation from the sharp corner of the indenter to the boundary of the crystalline and amorphous phases. **b** SEM image showing Pt deposition location (circled markers) during the FIB process for site selective TEM lamella preparation. **c** SEM image of the TEM lamella lift-out from the crystalline-amorphous interface, where the crack is arrested. **d** Cross sectional TEM micrograph of the interface region, showing that the crack is arrested by the amorphous zone (marked by the white dashed line)

As one example to demonstrate the unique mechanical properties of the heterogeneous structure having a patterned phase, micron indentation was performed to introduce local fracture. As shown in Fig. 5a, indentation on the crystalline region, which corresponds to the region protected by the TEM grid in Fig. 4a, induces a crack from the indenter corner. The crack propagates and intercepts the crystalline-amorphous boundary. For site selective characterization, the intercept location was marked by Pt deposition (Fig. 5b). A focused ion beam (FIB) lift-out technique was used to prepare TEM lamella from this location (Fig. 5c). A propagating crack is visible. A TEM micrograph shows that the propagating crack stops at the crystalline-amorphous interface.

The indentation-induced cracking in the crystalline region suggests embrittlement due to nanocrystallization. Previous studies have shown both toughening and embrittlement in partially crystallized MGs, depending on the effects of volume fraction, nanocrystal size, and nanocrystal mechanical properties. For a Zr-based MG, embrittlement was found to occur if the crystal volume fraction exceeds 6% [12]. Simulations show that hard nanocrystals can embrittle a MG while soft nanocrystals toughen a MG, with both effects increasing with increasing volume fraction of nanocrystals. Hard nanocrystals block shear band propagation, result in narrower shear band widths and promote local cavitation. Such embrittlement of the crystalline region, however, can be reduced by performing low fluence ion irradiation, after thermal annealing, to modify the crystal volume fraction. It is feasible to combine ion irradiation and masking to modify the volume fractions in both crystalline and amorphous regions to optimize the overall mechanical behavior of the heterogeneous structure.

It is possible to prepare masks of a few nanometer resolution based on the maturity of nanotechnology, bringing additional benefit from the size effect [30]. Earlier studies on crystalline-amorphous nanolaminates, fabricated by physical deposition, have shown a significant ductility increase and improved homogeneity in flow, contributing to both the size effect and interface effect [31]. When the amorphous layer thickness is less than a certain critical size, it becomes difficult for shear transformation zones to develop into mature shear bands, in terms of the aged-rejuvenation-glue-liquid shear band model [31]. The crystalline-amorphous interface, on the other hand, can absorb dislocations created in the crystalline region, leading to high strength nanolaminates.

The proposed procedure for phase patterning of MGs can find various applications. For example, for a Fe-based MG, it is possible to fabricate ordered nanocrystals with a given ferromagnetic property in an amorphous matrix, as a data storage device. Another application could be radiation detection, based on the resistivity difference between crystalline and amorphous phases [32]. Using polycrystalline MGs (by performing thermal annealing) as the starting material, the radiation dose of heavy ions or neutrons could be quantitatively determined by measuring resistivity changes. According to the present study, such phase change occurs up to a temperature close to T_g_. This opens a new door for radiation detection from room temperature to T_g_. It is feasible to use boron containing MGs to induce nuclear reactions with neutrons, for improving detection efficiency. It is worthy to note that electrical resistivity measurement for radiation dosage determination is difficult for traditional metals, e.g., pure Fe, due to very complicated defect clustering processes [33]. Point defect interactions and extended defect formation under defect supersaturation conditions lead to multiple stages of quasi-steady states of point defect populations, in which resistivity readings are challenging to interpret due to their complicated dependence on both temperature and time [33]. This makes it impossible to quantitatively determine the doses. Different from traditional metals, upon irradiation there is no recovery or reverse reaction in crystalline-to-amorphous phase changes. Hence, a linear volume change of amorphous phase is expected as a function of ion fluence.

## Conclusions

To conclude, we have shown that ion irradiation of an amorphous MG cannot induce phase changes in the damage cascade regions, because the quenching of the thermal spike and local melting zone is too fast. Ion irradiation stabilizes the amorphous phase even when irradiation is performed at temperatures slightly higher than the T_g_. Ion irradiation of an amorphous MG can create a heterogeneous structure, but over a very narrow temperature window (T_g_ + ~ 20 °C, in the present study). On the other hand, ion irradiation of an annealed MG can create patterned heterogeneous structures over a much larger temperature window (T < ~ T_g_). The key in the latter approach is to control the grain size to be comparable to the size of the damage cascade. Therefore, atomic mixing and local melting in the damage cascade regions create a liquid MG with a composition close to the original MG. For large grain sizes, it is difficult to average neighboring domains to achieve the original MG composition. Thus, critical cooling rates for MG formation in large crystalline domains are dramatically increased to allow crystallization upon quenching.

Applying the technique of thermal annealing and subsequent masked ion irradiation, we created patterned heterogeneous structures, with the damage cascade reachable region converted to an amorphous phase, and un-irradiated regions sustained as a crystalline phase. Indentation tests show that cracks created in the crystalline phase are arrested at the amorphous-crystalline interface. With the capability to further adjust crystal size, density and volume fraction in each patterned region, the mechanical properties of the heterogeneous structures can be further tuned and optimized. These findings also open new doors for various applications from data storage to radiation detection.

## Methods

### Specimen synthesis and preparation

A Ti_40_Cu_29_Zr_10_Pd_14_Sn_2_Si_5_ metallic glass ribbon, ~ 2 mm wide and ~ 30 μm thick was fabricated by melting mixtures of pure metal powders and fast quenching and solidification of the melt on a copper roller in an argon atmosphere. The study involves irradiation of both as-spun specimens and specimens after vacuum annealing at 600 and 800 °C for 2 and 4 h, respectively. The vacuum during annealing was about 1 × 10^−7^ torr.

### Modeling

The damage production by 3.5 MeV Cu ions was simulated by using the Stopping and Range of Ions in Matter (SRIM) code under the full damage cascades model [34]. The displacement energies of 30 eV (Ti), 30 eV (Cu), 25 eV (Pd), 40 eV (Zr), 60 eV (Nb), 25 eV (Sn), and 15 eV (Si) were selected [35]. The damage cascade thermal annealing was simulated by using the SRIM displacement distributions as input in a commercial finite element analysis code ANSYS [36]. The temperature of each heating source at time zero was converted from the interstitials’ kinetic energies, with a locally redefined mesh. A thermal conductivity of 5 W/mK [37], and specific heat of 0.383 kJ/kgK were used [38]. For the side facing the beam, thermal flux is set to zero. For all other sides, temperature is fixed to room temperature.

### Ion irradiation

Ion irradiation was performed using a General Ionex 1.7 MV tandetron accelerator located at Texas A&M University. The beam current was intentionally controlled to 70 nA to minimize beam heating. A thermocouple was attached over the target heater surface to monitor temperature. The overall temperature fluctuation over the irradiation was less than ± 5 °C. A slow raster beam with a spot size of 12 mm×6.5 mm was used. The vacuum during irradiation was 6 × 10^−7^ to 8 × 10^−7^ torr. A beam deflection technique was used to filter carbon and oxygen contamination [39, 40].

### Specimen characterization

A Bruker-D8 Discover X-ray diffractometer (XRD) with Cu Kα radiation (λ = 0.1540562 nm) was used to characterize the specimens. TEM lamellas were prepared by using a lift-out technique on a Tescan Lyra-3, and TEM characterization was performed by using an FEI Tecnai F20. For indentation, a Hysitron TI 950 Triboindenter was used with a Berkovich indenter at a maximum load of 3 N. A trapezoidal load function was used with a 5 s load – 2 s hold – 5 s unload.

### Supplementary information

**Additional file 1: Fig. S1.** **a** Three dimensional distribution of displacements by one 3.5 MeV Cu ion bombardment in a Ti_40_Cu_29_Zr_10_Pd_14_Sn_2_Si_5_ MG at room temperature, calculated by using the SRIM code. **b** Building initial ANSYS simulation with redefined mesh (purple) for high accurate temperature simulation. **Fig. S2.** Temperature evolution of the damage cascade region caused by one 3.5 MeV Cu ion bombardment in a Ti_40_Cu_29_Zr_10_Pd_14_Sn_2_Si_5_ MG at room temperature. **a** At time 50 ps. **b** 150 ps. **c** 250 ps. **d** 750 ps. Determined by a temperature boundary of 980 °C, the melting temperature of a Ti-Cu system, the melting zone is about 30 nm to 50 nm, varying among different damage cascades.** Fig. S3.** Scanning TEM (STEM) image and EDS line scan of a Ti_40_Cu_29_Zr_10_Pd_14_Sn_2_Si_5_ MG after irradiation at 440 °C. Pt is deposited during the FIB process. The red dashed line refers to the MG surface. The EDS line scan suggests there is Cu loss and Ti enrichment in the near surface region, from the surface to a depth of about 100 nm. **Fig. S4.** Cross sectional TEM micrograph of a MG after annealing at 800 °C for 4 hours to form large grains and then irradiated by 3.5 MeV Cu ions at room temperature. The two insets show HRTEM micrographs and diffraction patterns collected within the R_p_. The majority of the characterized regions show crystalline phases. A few local regions show an amorphous phase, which is attributed to an atom mixing effect under high fluence irradiation. **Fig. S5.** X-ray diffraction analysis of a MG after annealing at 600 °C for 2 h and subsequent Cu ion irradiation. The resulting crystalline phases are a combination of face-centered cubic Ti and two intermetallic compounds (body-centered tetragonal Cu_4_Ti_3_ and hexagonal TiPd_3_)

## Data Availability

The datasets used and/or analysed during the current study are available from the corresponding author on reasonable request.

## References

[1] Peker A, Johnson WL (1993). A highly processable metallic glass: Zr_41.2_Ti_13.8_Cu_12.5_Ni_10.0_Be_22.5_. Appl. Phys. Lett..

[2] Greer AL (1995). Metallic glasses. Science.

[3] Inoue A (2000). Stabilization of metallic supercooled liquid and bulk amorphous alloys. Acta Mater..

[4] Wang WH, Dong C, Shek CH (2004). Bulk metallic glasses. Mater. Sci. Eng. R-Rep..

[5] Hofmann DC, Suh J-Y, Wiest A, Duan G, Lind M-L, Demetriou MD, Johnson WL (2008). Designing metallic glass matrix composites with high toughness and tensile ductility. Nature.

[6] Gilbert CJ, Ritchie RO, Johnson WL (1997). Fracture toughness and fatigue-crack propagation in a Zr-Ti-Ni-Cu-Be bulk metallic glass. Appl. Phys. Lett..

[7] Nagendra N, Ramamurty U, Goh TT, Li Y (2000). Effect of crystallinity on the impact toughness of a La-based bulk metallic glass. Acta Mater..

[8] Kumar G, Rector D, Conner RD, Schroers J (2009). Embrittlement of Zr-based bulk metallic glasses. Acta Mater..

[9] Raghavan R, Shastry VV, Kumar A, Jayakumar T, Ramamurty U (2009). Toughness of as-cast and partially crystallized composites of a bulk metallic glass. Intermetallics.

[10] Song M, Li Y, Wu Z, He Y (2011). The effect of annealing on the mechanical properties of a ZrAlNiCu metallic glass. J. Non-Cryst. Solids.

[11] Kong J, Ye Z, Li J, Chen W, Ma X (2012). Embrittlement of a bulk metallic glass containing ductile phase after low-temperature annealing. Phys. Status Solidi B.

[12] Ketkaew J, Liu Z, Chen W, Schroers J (2015). Critical crystallization for embrittlement in metallic glasses. Phys. Rev. Lett..

[13] Deng B, Shi Y (2019). The embrittlement and toughening of metallic glasses from nano-crystallization. J Appl. Phys..

[14] Lu K, Wang JT (1991). Activation energies for crystal nucleation and growth in amorphous alloys. Mater. Sci. Eng..

[15] Lu W, Yan B, Huang W-H (2005). Complex primary crystallization kinetics of amorphous Finemet alloy. J. Non-Cryst. Solids.

[16] Xu T, Jian Z, Chang F, Zhuo L, Zhang T (2018). Non-isothermal crystallization kinetics of Fe_75_Cr_5_P_9_B_4_C_7_ metallic glass with a combination of desired merits. Vacuum.

[17] Chen H, He Y, Shiflet J, Poon SJ (1994). Deformation-induced nanocrystal formation in shear bands of amorphous alloys. Nature.

[18] Trudeau ML, Schulz R, Dussault D, Van Neste A (1990). Structural changes during high-energy ball milling of iron-based amorphous alloys: is high-energy ball milling equivalent to a thermal process?. Phys. Rev. Lett..

[19] Kim J-J, Choi Y, Suresh S, Argon AS (2002). Nanocrystallization during nanoindentation of a bulk amorphous metal alloy at room temperature. Science.

[20] Wang ZX, Li FY, Pan MX, Zhao DQ, Wang WH (2005). Effects of high pressure on the nucleation of Cu_60_Zr_20_Hf_10_Ti_10_ bulk metallic glass. J. Alloys Comp..

[21] Nagase T, Umakoshi Y (2005). Thermal crystallization and electron irradiation induced phase transformation behavior in Zr_66.7_Cu_33.3_ metallic glass. Mater. Trans..

[22] Carter J, Fu EG, Martin M, Xie G, Zhang X, Wang YQ, Wijesundera D, Wang XM, Chu W-K, McDeavitt SM, Shao L (2009). Ion irradiation induced nanocrystal formation in amorphous Zr_55_Cu_30_Al_10_Ni_5_ alloy. Nucl. Inst. Meth Phys. Res. B.

[23] Fu EG, Carter J, Martin M, Xie G, Zhang X, Wang YQ, Littleton R, Shao L (2009). Electron irradiation-induced structural transformation in metallic glasses. Scripta Mater..

[24] Raghavan R, Kombaiah B, Döbeli M, Erni R, Ramamurty U, Michler J (2012). Nanoindentation response of an ion irradiated Zr-based bulk metallic glass. Mat. Sci. Eng. A.

[25] Spaepen F (1977). A microscopic mechanism for steady state inhomogeneous flow in metallic glasses. Acta Metall..

[26] Rosato V, Cleri F (1992). A molecular dynamics simulation of the effects of excess free volume on the diffusion in metallic glasses. J. Non-Crystal Solids.

[27] Myers M, Fu EG, Myers M, Wang H, Xie G, Wang X, Chu W-K, Shao L (2010). An experimental and modeling study on the role of damage cascade formation in nanocystallization of ion-irradiated Ni_52.5_Nb_10_Zr_15_Ti_15_Pt_7.5_ metallic glass. Scripta Mater..

[28] Suer S, Mekhrabov AO, Vedat Akdeniz M (2009). Theoretical prediction of bulk glass forming ability (BGFA) of Ti-Cu based multicomponent alloys. J. Non-cryst. Solids.

[29] Shao L, Chen D, Zare A, Lucca DA (2021). Mechanism of nanocrystallization temperature shifting during ion irradiation of metallic glasses. Nucl. Instrum. Methods Phys. Res. B.

[30] Shimizu F, Ogata S, Li J (2006). Yield point of metallic glass. Acta Mater..

[31] Wang Y, Li J, Hamza AV, Barbee TW (2007). Jr. Ductile crystalline–amorphous nanolaminates. PNAS.

[32] Mitra A, Rao V, Pramanik S, Mohanty ON (1992). Crystallization study of amoprhous Fe_40_Ni_40_B_20_ by electrical resistivty measurement. J. Mater. Sci..

[33] Fu C-C, Torre JD, Willaime F, Bocquet J-L, Barbu A (2005). Multiscale modeling of defect kinetic in irradiated iron. Nat. Mater..

[34] J. Ziegler, *The Stopping and Range of Ions in Solids, Ion Implantation: Science and Technology*, Second edn. (Elsevier, 1984), pp. 3–61

[35] Standard practice for, neutron radiaiton damage simulatin by charged-particle irradiation, (American Soceity for Testing and Materials, ASTM-E521-96, Philadelphia West Conshohocken, 1966)

[36] ANSYS Fluent user, *s guide, release 17.2* (ANSYS, Inc., Canonsburg, Pennsylvania, 2016)

[37] Kuo YK, Sivakumar KM, Su CA, Ku CN, Lin ST, Kaiser AB, Qiang JB, Wang Q, Dong C (2006). Measurement of low-temperature transport properties of Cu-based Cu-Zr-Ti bulk metallic glass. Phys. Rev. B.

[38] Wang G, Huang Y-J, Makhanlall D, Shen J (2017). Resistance spot welding of Ti_40_Zr_25_Ni_3_Cu_12_Be_20_ bulk metallic glass: experiments and finite element modeling. Rare Met..

[39] Shao L, Gigax J, Chen D, Kim H, Garner FA, Wang J, Toloczko MB (2017). Standardization of accelerator irradiation procedures for simulation of neutron induced damage in reactor structural materials. Nucl. Inst. Meth Phys. Res. B.

[40] Gigax J, Kim H, Aydogan E, Price LM, Wang X, Maloy SA, Garner FA, Shao L (2019). Impact of composition modification induced by ion beam Coulomb-drag effects on the nanoindentation hardness of HT9. Nucl. Inst. Meth Phys. Res. B.

