# Supplementary material for: Phase patterning of metallic glasses through superfast quenching of ion irradiation-induced thermal spikes
Source: Nano Converg. 2023 Nov 21;10:54. doi: 10.1186/s40580-023-00400-7 (PMC10663422; doi:10.1186/s40580-023-00400-7)
Supplement: Supplementary file 1 — Additional file 1: Fig. S1. a Three dimensional distribution of displacements by one 3.5 MeV Cu ion bombardment in a Ti40Cu29Zr10Pd14Sn2Si5 MG at room temperature, calculated by using the SRIM code. b Building initial ANSYS simulation with redefined mesh (purple) for high accurate temperature simulation. Fig. S2. Temperature evolution of the damage cascade region caused by one 3.5 MeV Cu ion bombardment in a Ti40Cu29Zr10Pd14Sn2Si5 MG at room temperature. a At time 50 ps. b 150 ps. c 250 ps. d 750 ps. Determined by a temperature boundary of 980 °C, the melting temperature of a Ti-Cu system, the melting zone is about 30 nm to 50 nm, varying among different damage cascades. Fig. S3. Scanning TEM (STEM) image and EDS line scan of a Ti40Cu29Zr10Pd14Sn2Si5 MG after irradiation at 440 °C. Pt is deposited during the FIB process. The red dashed line refers to the MG surface. The EDS line scan suggests there is Cu loss and Ti enrichment in the near surface region, from the surface to a depth of about 100 nm. Fig. S4. Cross sectional TEM micrograph of a MG after annealing at 800 °C for 4 hours to form large grains and then irradiated by 3.5 MeV Cu ions at room temperature. The two insets show HRTEM micrographs and diffraction patterns collected within the Rp. The majority of the characterized regions show crystalline phases. A few local regions show an amorphous phase, which is attributed to an atom mixing effect under high fluence irradiation. Fig. S5. X-ray diffraction analysis of a MG after annealing at 600 °C for 2 h and subsequent Cu ion irradiation. The resulting crystalline phases are a combination of face-centered cubic Ti and two intermetallic compounds (body-centered tetragonal Cu4Ti3 and hexagonal TiPd3) [file 40580_2023_400_MOESM1_ESM.docx]

**Supplementary documents**

**Phase patterning of metallic glasses through superfast quenching of ion irradiation-induced thermal spikes**

Hyosim Kim^1^, Tianyao Wang^2^, Jonathan Gigax^1^, Arezoo Zare^3^, Don A. Lucca^3^, Zhihan Hu^2^, Yongchang Li^2^, Trevor Parker^2^, Lin Shao^2,*^

*^1^ Los Alamos National Laboratory, Los Alamos, NM 87545, USA*

*^2^ Department of Nuclear Engineering, Texas A&M University, College Station, TX 77843, USA*

*^3^ School of Mechanical and Aerospace Engineering, Oklahoma State University, Stillwater, OK 74078, USA*


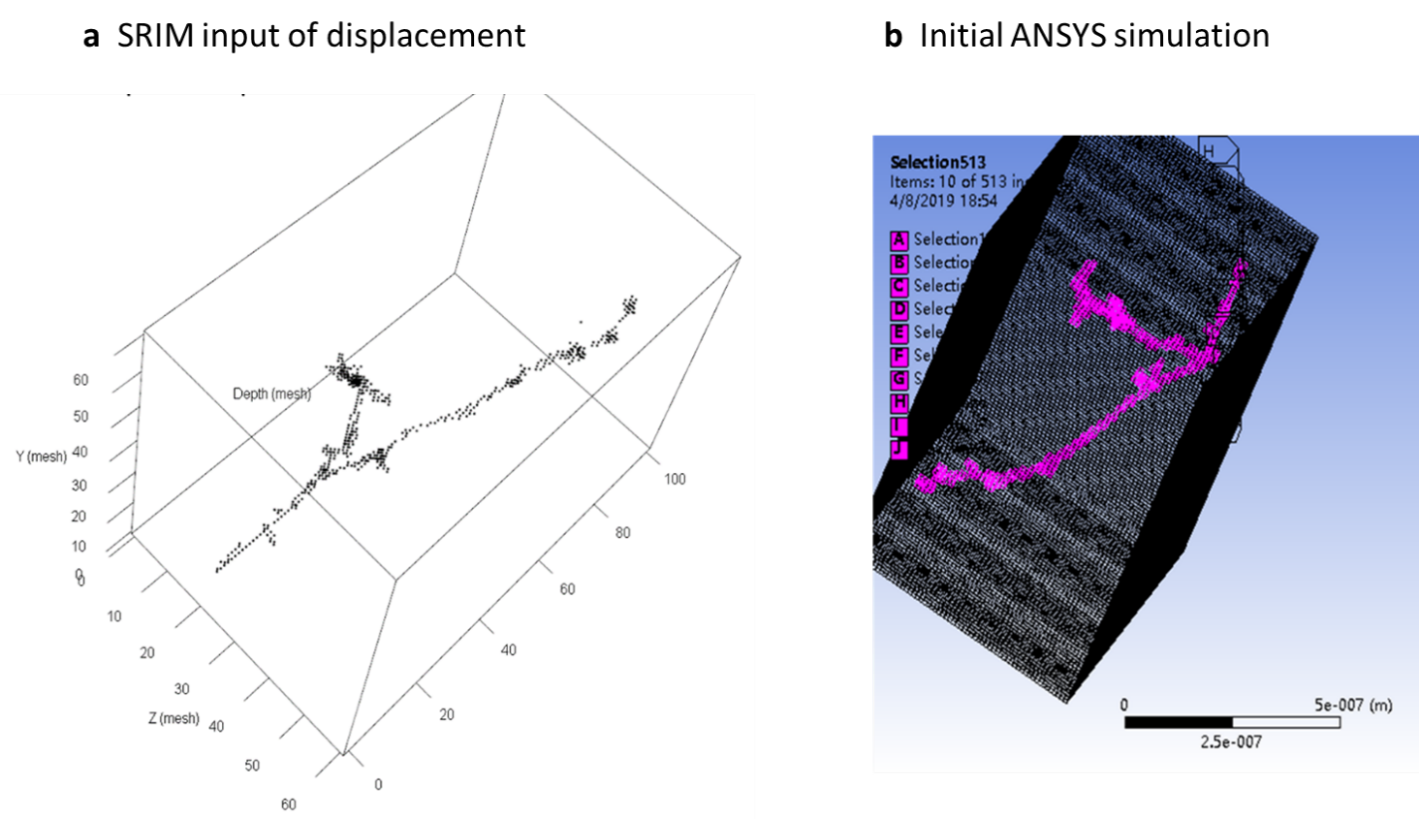


Fig. S1 **a.** Three dimensional distribution of displacements by one 3.5 MeV Cu ion bombardment in a Ti_40_Cu_29_Zr_10_Pd_14_Sn_2_Si_5_ MG at room temperature, calculated by using the SRIM code. **b.** Building initial ANSYS simulation with redefined mesh (purple) for high accurate temperature simulation.


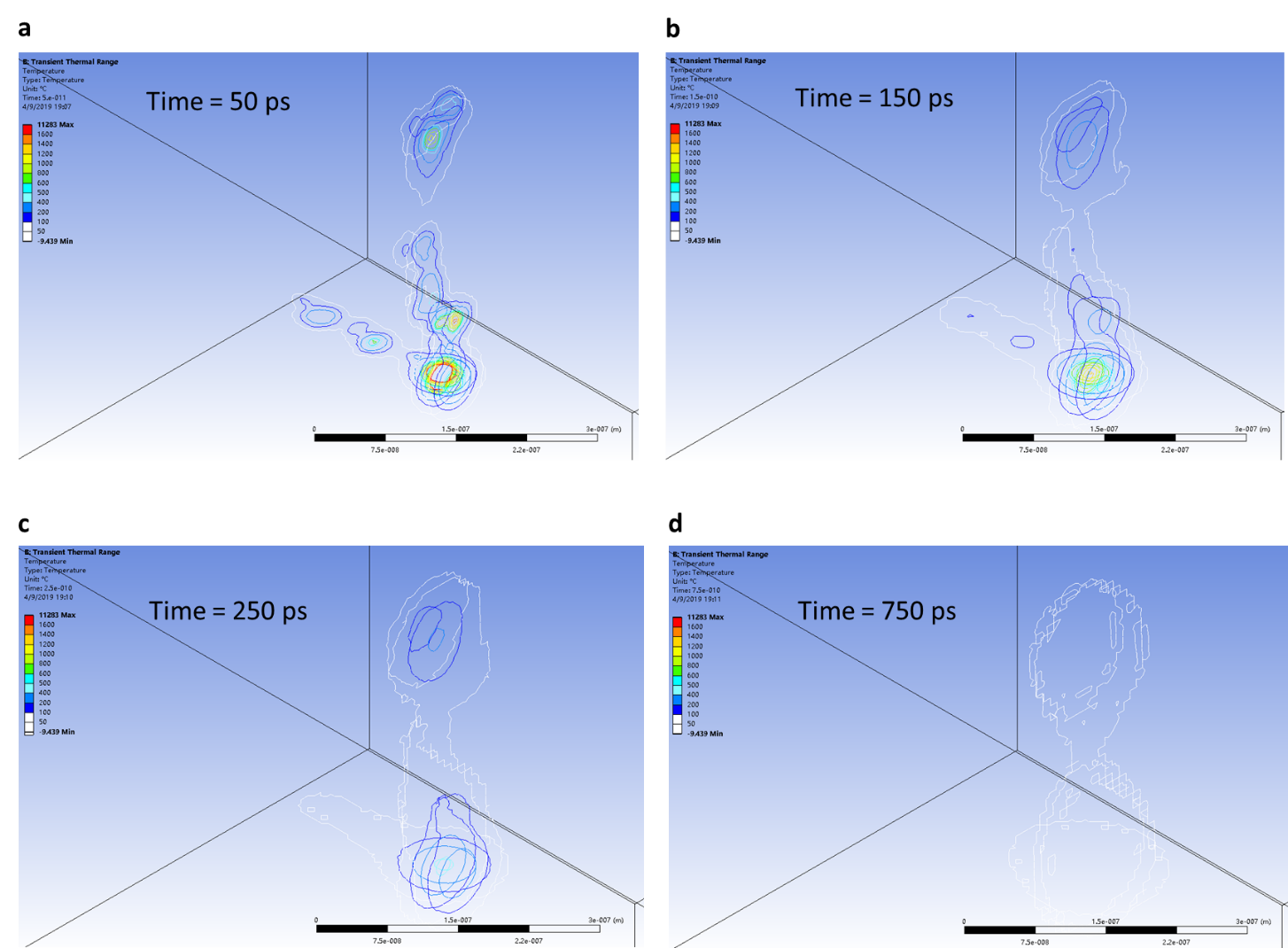


Fig. S2 Temperature evolution of the damage cascade region caused by one 3.5 MeV Cu ion bombardment in a Ti_40_Cu_29_Zr_10_Pd_14_Sn_2_Si_5_ MG at room temperature. **a.** At time 50 ps. **b.** 150 ps. **c.** 250 ps. **d.** 750 ps. Determined by a temperature boundary of 980 °C, the melting temperature of a Ti-Cu system, the melting zone is about 30 nm to 50 nm, varying among different damage cascades.


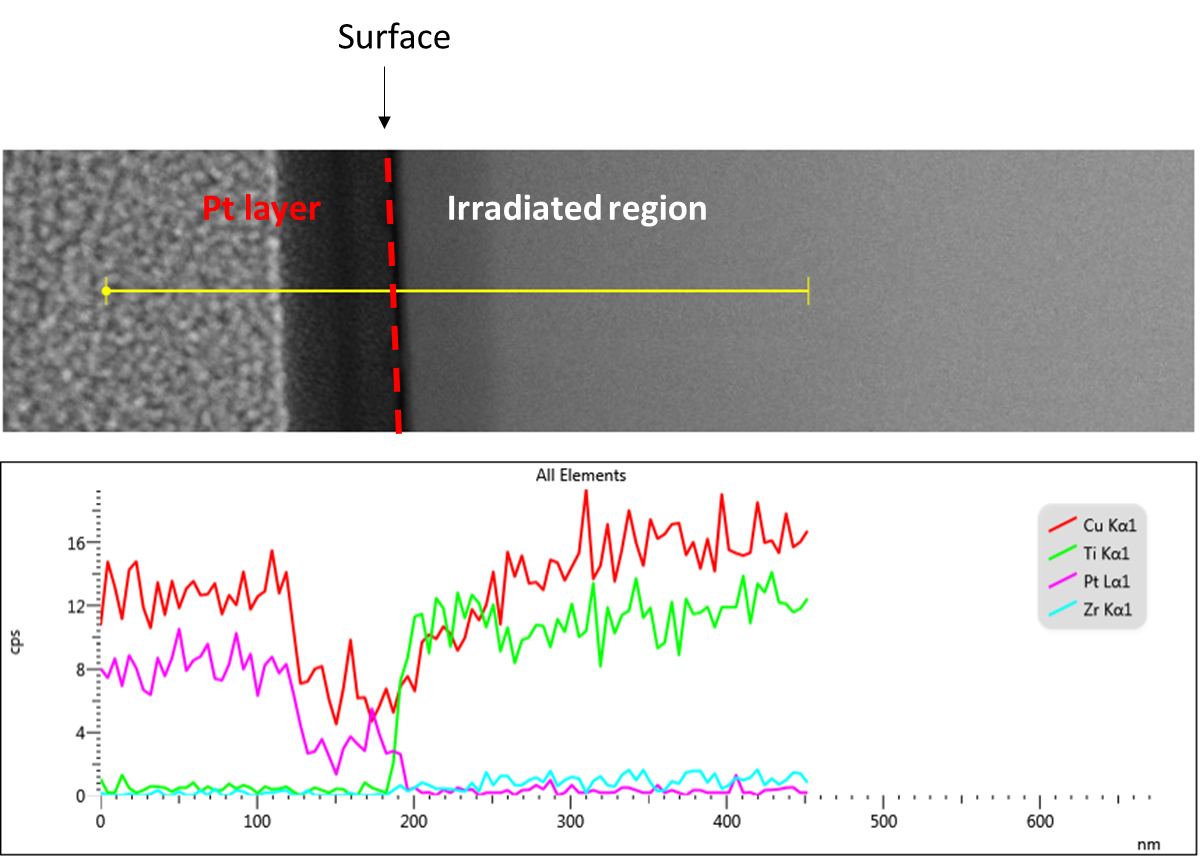


Fig. S3 Scanning TEM (STEM) image and EDS line scan of a Ti_40_Cu_29_Zr_10_Pd_14_Sn_2_Si_5_ MG after irradiation at 440 °C. Pt is deposited during the FIB process. The red dashed line refers to the MG surface. The EDS line scan suggests there is Cu loss and Ti enrichment in the near surface region, from the surface to a depth of about 100 nm.


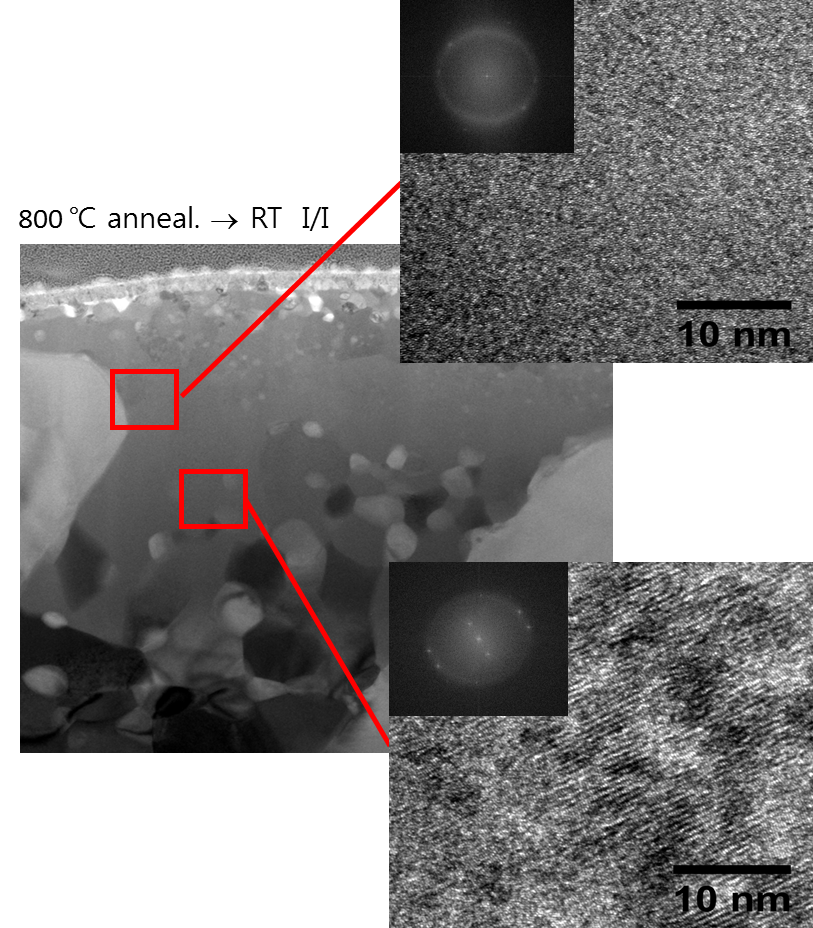


Fig. S4 Cross sectional TEM micrograph of a MG after annealing at 800 °C for 4 hours to form large grains and then irradiated by 3.5 MeV Cu ions at room temperature. The two insets show HRTEM micrographs and diffraction patterns collected within the Rp. The majority of the characterized regions show crystalline phases. A few local regions show an amorphous phase, which is attributed to an atom mixing effect under high fluence irradiation.


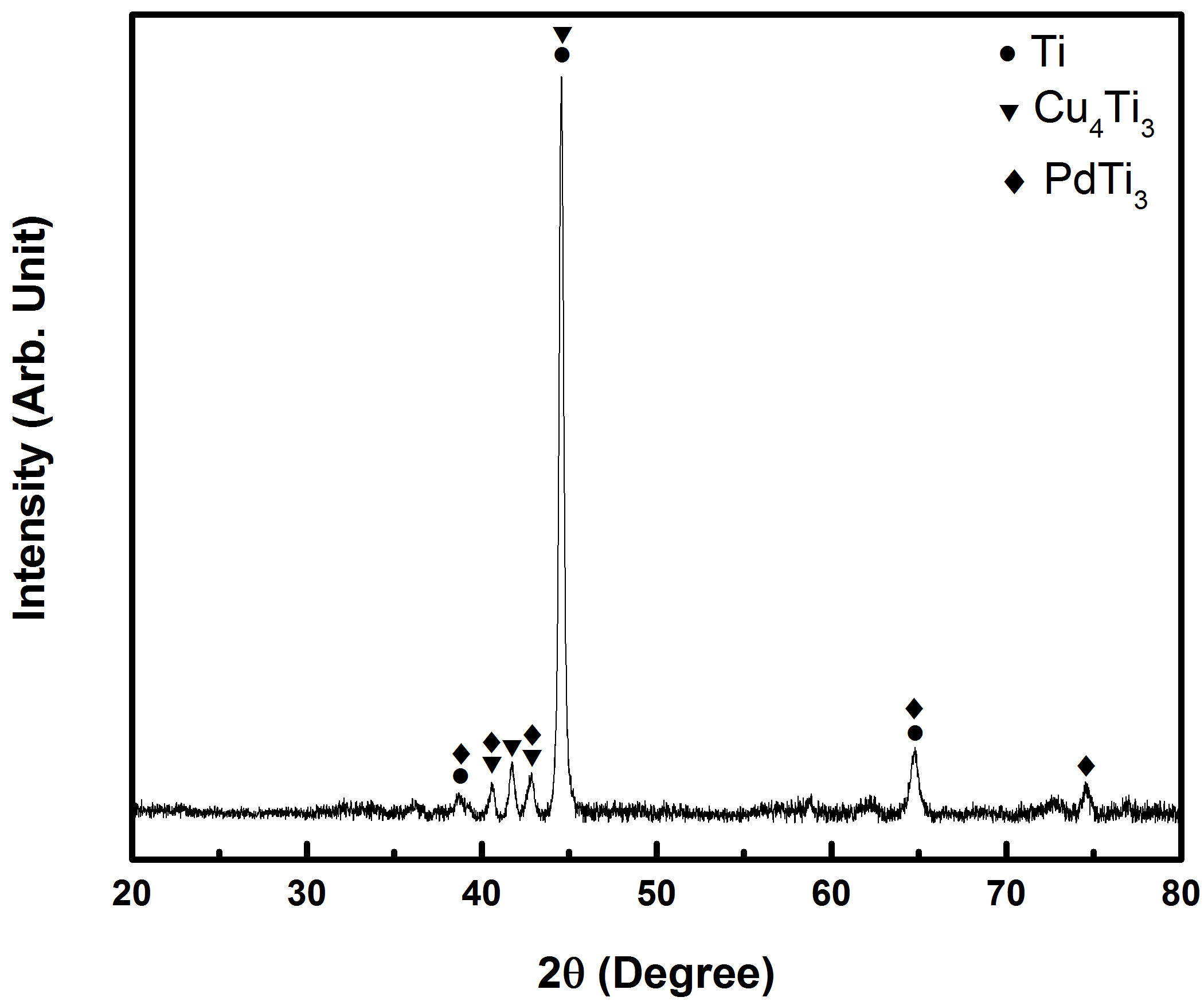


Fig. S5 X-ray diffraction analysis of a MG after annealing at 600 °C for 2 hours and subsequent Cu ion irradiation. The resulting crystalline phases are a combination of face-centered cubic Ti and two intermetallic compounds (body-centered tetragonal Cu_4_Ti_3_ and hexagonal TiPd_3_).
